# Intracellular Mg^2+^ protects mitochondria from oxidative stress in human keratinocytes

**DOI:** 10.1038/s42003-023-05247-6

**Published:** 2023-08-24

**Authors:** Keigo Fujita, Yutaka Shindo, Yuji Katsuta, Makiko Goto, Kohji Hotta, Kotaro Oka

**Affiliations:** 1 Department of Bioscience and Informatics, Faculty of Science and Technology, Keio University, Yokohama, Japan; 2 School of Frontier Engineering, Kitasato University, Sagamihara, Japan; 3 MIRAI Technology Institute, Shiseido Co. Ltd., Yokohama, Japan; 4 Waseda Research Institute for Science and Engineering, Waseda University, Tokyo, Japan; 5 Graduate Institute of Medicine, College of Medicine, Kaohsiung Medical University, Kaohsiung City, Taiwan

**Keywords:** Stress signalling, Cellular imaging

## Abstract

Reactive oxygen species (ROS) are harmful for the human body, and exposure to ultraviolet irradiation triggers ROS generation. Previous studies have demonstrated that ROS decrease mitochondrial membrane potential (MMP) and that Mg^2+^ protects mitochondria from oxidative stress. Therefore, we visualized the spatio-temporal dynamics of Mg^2+^ in keratinocytes (a skin component) in response to H_2_O_2_ (a type of ROS) and found that it increased cytosolic Mg^2+^ levels. H_2_O_2_-induced responses in both Mg^2+^ and ATP were larger in keratinocytes derived from adults than in keratinocytes derived from newborns, and inhibition of mitochondrial ATP synthesis enhanced the H_2_O_2_-induced Mg^2+^ response, indicating that a major source of Mg^2+^ was dissociation from ATP. Simultaneous imaging of Mg^2+^ and MMP revealed that larger Mg^2+^ responses corresponded to lower decreases in MMP in response to H_2_O_2_. Moreover, Mg^2+^ supplementation attenuated H_2_O_2_-induced cell death. These suggest the potential of Mg^2+^ as an active ingredient to protect skin from oxidative stress.

## Abstract (web summary)

Visualization of Mg^2+^ dynamics in keratinocytes shows that a larger Mg^2+^ response corresponds to lower decreases of mitochondrial membrane potential in response to oxidative stress, suggesting a protective role for Mg^2+^ on mitochondria.

## Introduction

Reactive oxygen species (ROS) are constantly produced in the human body and have harmful effects. Exposure to ultraviolet (UV) irradiation in particular is a notable trigger for ROS generation^1^. ROS generation is attributed to several factors represented by enzyme activities of the electron transport chain in mitochondria^2^. Oxidative stress induced by ROS constitutes a harmful condition because ROS have high reactivity and cause DNA damage, lipid peroxidation, and protein carbonylation. These forms of oxidative damage increase with age^3,4^. Excessive ROS generation also causes mitochondrial dysfunction. In cellular-level experiments, the addition of H_2_O_2_ (a type of ROS) decreased mitochondrial membrane potential (MMP)^5,6^. Moreover, it has been reported that ROS-induced mitochondrial dysfunction contributes to various diseases, including Alzheimer’s disease, type 1 diabetes, atherosclerosis, and cancer^7^. Skin in particular is frequently exposed to ROS stress since UV irradiation from sunlight is the main generator of ROS^1^; ROS stress has been suggested to be involved in aging, inflammation, and pathogenesis of skin cancer, among other conditions^8,9^. Therefore, protecting biomolecules and mitochondria from ROS is critical for maintaining normal cellular functions, and cells express antioxidants and several mechanisms to avoid oxidative stress^10^. Skin cells, such as the keratinocytes and fibroblasts which constitute the epidermis, also have ROS clearance mechanisms^11^. In our previous study, we suggested that Mg^2+^ was involved in mechanisms to help cells avoid oxidative stress^12^.

Mg^2+^ is the most abundant divalent cation in living cells and is related to more than 600 enzymes as a cofactor^13–15^. Recent studies have demonstrated that small changes in intracellular Mg^2+^ can have large impacts on cellular events, such as cell division, maturation of neurons, and neurodegeneration^16–18^. In neurons and cancer cells, intracellular Mg^2+^ is stored in the mitochondria and constitutes Mg^2+^ an important factor for sustainable mitochondrial functioning^12,19^. The impact of Mg^2+^ in relation to retaining MMP has been reported at the cellular and isolated mitochondrial levels^20–22^. The protective effects of Mg^2+^ toward oxidative stress have also been reported in various types of cells, such as endothelial cells^23,24^, bone marrow mesenchymal stem cells^25,26^, and chick embryo hepatocytes^27^. On the other hand, in monocytes the environment is the determining factor in whether high levels of Mg^2+^ will increase or decrease ROS levels^28^. These findings clearly indicate the important role of Mg^2+^ in protecting cells from oxidative stress. However, it has not been well understood whether cells change intracellular Mg^2+^ concentrations in response to oxidative stress, nor exactly how Mg^2+^ protects cells from oxidative stress.

In this study, we examined changes in Mg^2+^ concentration in response to ROS in keratinocytes. Keratinocytes were chosen because they are one of the most ROS-exposed cells in the human body, as ROS are generated by UV from sunlight. The dynamics of cytosolic Mg^2+^ were visualized using fluorescence imaging under H_2_O_2_ stress to find that H_2_O_2_ induced an increase in cytosolic Mg^2+^ concentration ([Mg^2+^]_cyto_) due to the release of Mg^2+^ bound to ATP upon the H_2_O_2_-induced decrease in ATP level. The effects of Mg^2+^ on mitochondrial functions were investigated by comparison to changes in MMP to find that the increased Mg^2+^ attenuated the decrease in MMP. Interestingly, Mg^2+^ supplementation further suppressed the decrease in MMP and H_2_O_2_-induced cell death. Our results suggest that Mg^2+^ provides robustness to intracellular ATP levels under oxidative stress.

## Results

### H_2_O_2_-induced cytosolic Mg^2+^ increase

**Fig. 1:**
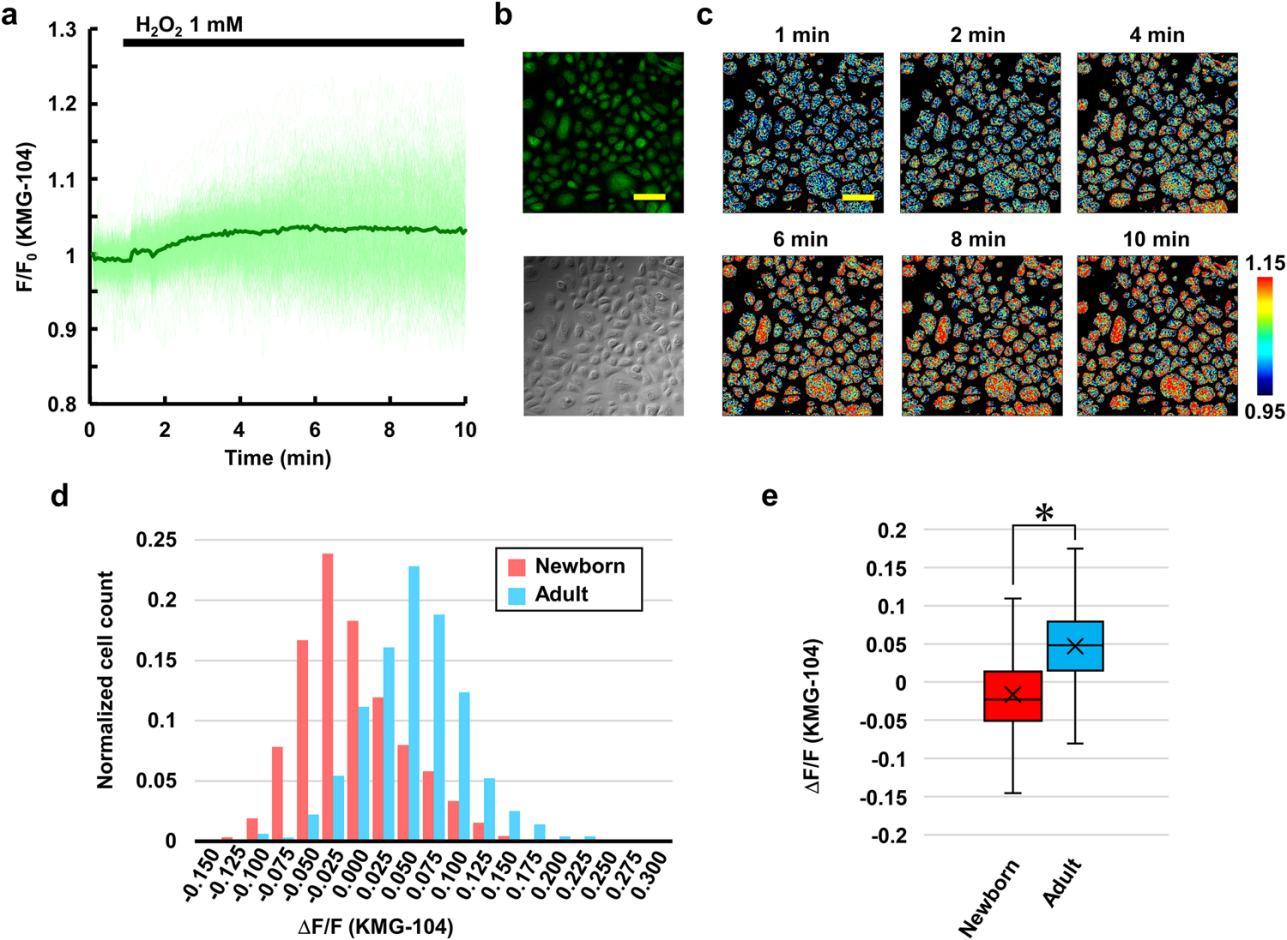
[Mg^2+^]_cyto_ increase in response to H_2_O_2_ in keratinocytes. **a** Time-course of [Mg^2+^]_cyto_ in response to H_2_O_2_ (1 mM) added at 1 min in adult keratinocytes. Mean (green line) and all traces (light green lines) (*n* = 511 cells from four different experiments). **b** Fluorescence image of keratinocytes stained with KMG-104 and differential interference contrast (DIC) image. **c** Pseudo-color image of Mg^2+^ dynamics (*F*/*F*_0_) in adult keratinocytes at the indicated time points. **d** Histogram showing the distribution of Mg^2+^ responses induced by H_2_O_2_ (1 mM) in newborn keratinocytes (red histogram: *n* = 1888 cells from nine different experiments, which include data from three keratinocyte cell lines; 0 years-1: *n* = 594 cells from three different experiments, 0 years-2: *n* = 598 cells from three different experiments, and 0 years-3: *n* = 696 cells from three different experiments) and adult keratinocytes (blue histogram: *n* = 994 cells from seven different dishes, which include data from two keratinocyte cell lines; 40 years: *n* = 511 cells from four different experiments, and 57 years: n = 483 cells from three different experiments). **e** Comparison of the average amplitude of Mg^2+^ response shown in **d**. The amplitude was calculated as a difference between the average of *F*/*F*_0_ before (0–1 min) and after (9–10 min) H_2_O_2_ treatment. Center line: median, *x*: average, box limits: quartiles, whiskers: 1.5× interquartile range. Scale bar in this figure: 100 μm. *p < 0.05 (Student’s *t*-test, two-sided).

Spatio-temporal dynamics of Mg^2+^ in keratinocytes derived from adults (adult keratinocytes) were visualized with an Mg^2+^ selective indicator, KMG-104. It was confirmed that H_2_O_2_ did not directly react with KMG-104 (Supplementary Fig. 1a). H_2_O_2_ caused the [Mg^2+^]_cyto_ to increase in keratinocytes from 40-year-old donor (Fig. 1a–c). The average time-course of [Mg^2+^]_cyto_ increased immediately after application of 1 mM H_2_O_2_ and reached a plateau at 5 min (Fig. 1a). The spatial distribution of the changes in [Mg^2+^]_cyto_ was almost uniform within the cells, whereas the amplitude of the change in each cell varied (Fig. 1c). To examine whether the H_2_O_2_-induced Mg^2+^ increase constituted a common phenomenon in keratinocytes across individual differences and age, the responses were compared in five keratinocyte cell lines: three from newborns (three different 0 years old donors: newborn keratinocytes) and two from adults (40 and 57 years old donors: adult keratinocytes). There was large variability in the amplitudes of the H_2_O_2_-induced Mg^2+^ response in each cell, regardless of age (Fig. 1d and Supplementary Fig. 2a). While some newborn keratinocytes increased and others decreased in [Mg^2+^]_cyto_ in response to H_2_O_2_ for all three cell lines, most of the adult keratinocytes showed increases in [Mg^2+^]_cyto_. As a result, all newborn keratinocyte lines showed no or slightly decreased [Mg^2+^]_cyto_ responses on average, whereas adult keratinocytes lines exhibited increased [Mg^2+^]_cyto_ on average (Supplementary Fig. 2b). Therefore, the data were divided into two groups, newborn and adult, and the difference in these two groups were compared. The amplitude of H_2_O_2_-induced Mg^2+^ responses was significantly larger in adult keratinocytes than in newborn keratinocytes (Fig. 1e). A higher concentration of H_2_O_2_ (10 mM) elicited increases in [Mg^2+^]_cyto_, even in newborn keratinocytes, indicating that this is not a phenomenon specific to adult keratinocytes, but simply that adult keratinocytes are more sensitive to H_2_O_2_ than newborn keratinocytes (Supplementary Fig. 2c, d). The responses to 10 mM H_2_O_2_ were also larger in adult keratinocytes than in newborn keratinocytes (Supplementary Fig. 2e, f). To examine underlying mechanism of the increase in [Mg^2+^]_cyto_ and role of the Mg^2+^, the following experiments were performed in adult keratinocytes from a 40-year-old donor.

### A major source of Mg^2+^ is Mg^2+^ dissociation from ATP in the process of ATP consumption

**Fig. 2:**
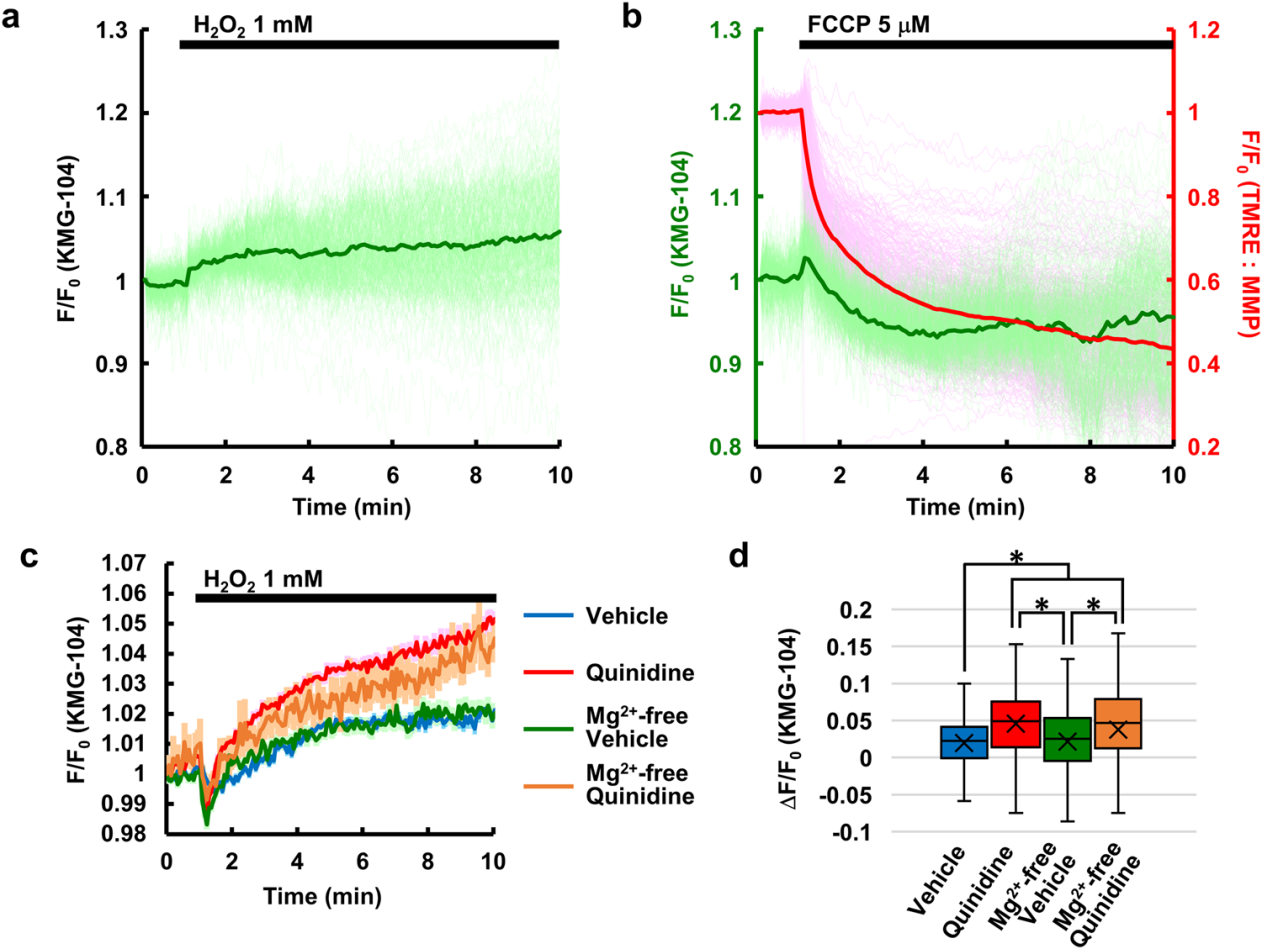
Examination of Mg^2+^ source in response to H_2_O_2_. **a** H_2_O_2_-induced Mg^2+^ response in Mg^2+^-free HBSS. Mean (green line) and all traces (light green lines) (*n* = 376 cells from three different experiments). **b** Time-courses of [Mg^2+^]_cyto_ (green line indicates mean and light green lines indicate all traces, left axis) and mitochondrial membrane potential (MMP) (red line indicates mean and pink lines indicate all traces, right axis), measured simultaneously, in response to FCCP (5 μM) added at 1 min (*n* = 394 cells from three different experiments). **c** Average time-courses of [Mg^2+^]_cyto_ in response to H_2_O_2_ in the presence of vehicle (0.5% DMSO; blue line, *n* = 512 cells from four different experiments), quinidine (200 μM; red line, *n* = 485 cells from four different experiments), vehicle in Mg^2+^-free condition (green line, *n* = 340 cells from three different experiments), and quinidine in Mg^2+^-free condition (orange, *n* = 389 cells from three different experiments). Error bars: SEM. **d** Comparison of the average amplitude of Mg^2+^ response shown in **c**. The amplitude was calculated as a difference between the average of *F*/*F*_0_ before (0–1 min) and after (9–10 min) H_2_O_2_ treatment. Center line: median, *x*: average, box limits: quartiles, whiskers: 1.5× interquartile range. *p < 0.05 (Tukey’s test, two-sided).

To identify the Mg^2+^ source that was responding to H_2_O_2_, we examined Mg^2+^ entry from an extracellular medium, and fluorescence imaging was performed in the medium without Mg^2+^. An H_2_O_2_-induced Mg^2+^ increase was still observed in this condition (Fig. 2a), which indicates that H_2_O_2_ induced the release of Mg^2+^ from an intracellular Mg^2+^ source. In other cell types, mitochondria were identified as intracellular Mg^2+^ storage sites and released Mg^2+^ into cytosol upon a depolarization of MMP^19,29,30^. Simultaneous imaging of [Mg^2+^]_cyto_ and MMP revealed that FCCP, an uncoupler of mitochondria, rapidly decreased MMP; however, FCCP did not increase [Mg^2+^]_cyto_ in keratinocytes, but rather deceased it (Fig. 2b). This indicates that depolarization of the mitochondria did not cause Mg^2+^ release from mitochondria in keratinocytes.

Next, the involvement of Mg^2+^ transporters was investigated. It has been reported that Na^+^/Mg^2+^ exchangers, one of which is SLC41A1, mediate Mg^2+^ efflux from the cells and are inhibited by quinidine^14,22,31^. Therefore, if one of the targets of H_2_O_2_ is the Na^+^/Mg^2+^ exchanger, which leads to the increase in [Mg^2+^]_cyto_, it is expected that quinidine also increases [Mg^2+^]_cyto_ and that prior application of quinidine abolishes the H_2_O_2_-induced increase in [Mg^2+^]_cyto_. Quinidine alone induced increase in [Mg^2+^]_cyto_ in both normal and Mg^2+^-free conditions, whereas vehicle (DMSO, final concentration 0.5%) decreased [Mg^2+^]_cyto_, indicating that inhibition of Mg^2+^ efflux leads to increase in [Mg^2+^]_cyto_ in keratinocytes (Supplementary Fig. 3a). The amplitude of quinidine-induced Mg^2+^ increase was greater in normal condition than Mg^2+^-free condition (Supplementary Fig. 3b). These results suggest that [Mg^2+^]_cyto_ is normally balanced by Mg^2+^ influx and efflux. The effect of quinidine on H_2_O_2_-induced Mg^2+^ responses was also investigated. The responses were greater in the presence of quinidine than that in the presence of vehicle both in normal and Mg^2+^-free medium (Fig. 2c, d), while those were slightly smaller in the presence of vehicle (DMSO, final concentration 0.5%) than in the absence of vehicle (compare Fig. 2a and c). This result indicates that the Na^+^/Mg^2+^ exchanger does not mediate H_2_O_2_-induced Mg^2+^ responses and that inhibition of Mg^2+^ efflux retains Mg^2+^ released from intracellular Mg^2+^ sources.

**Fig. 3:**
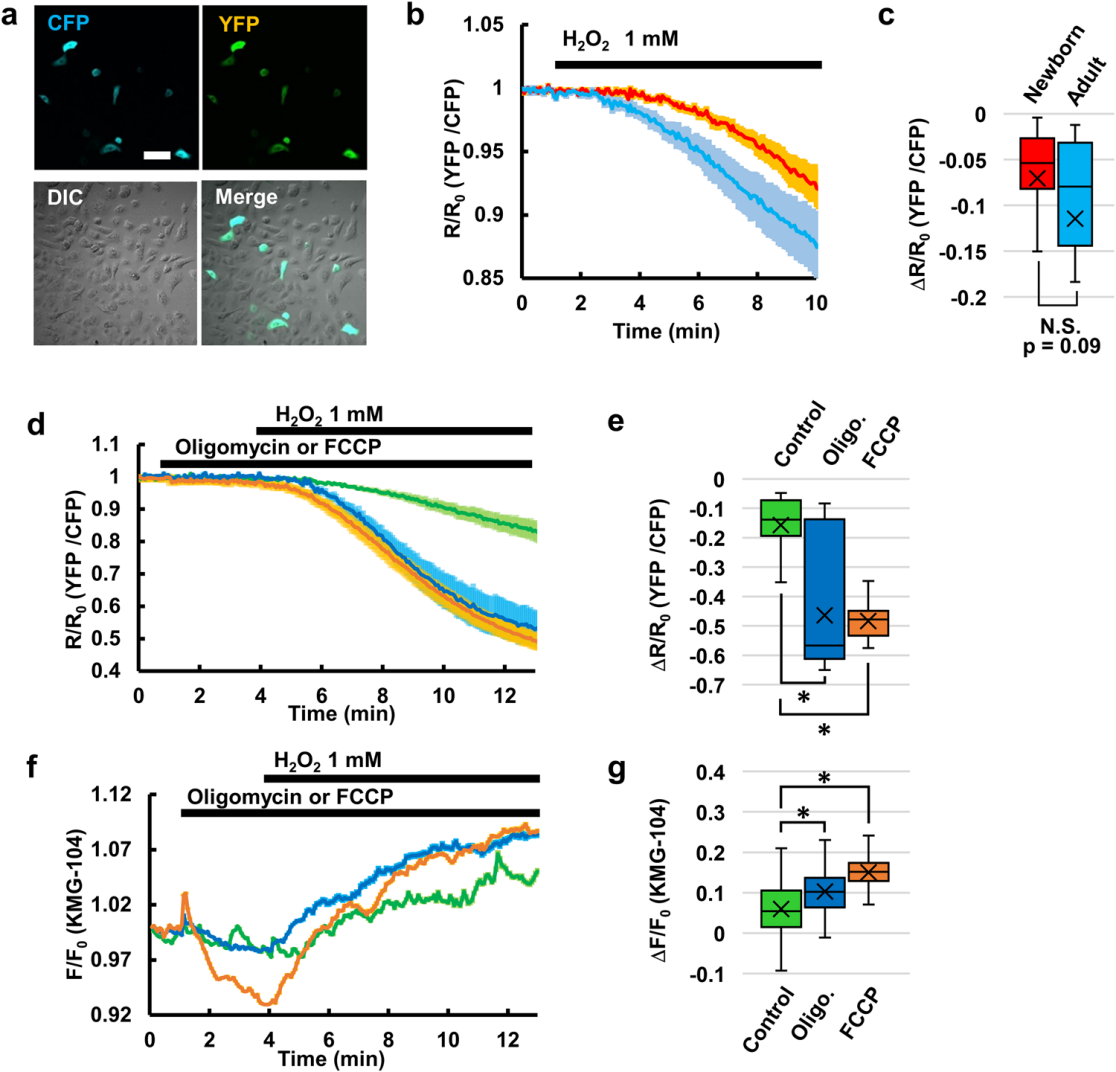
Inhibition of oxidative phosphorylation enhances H_2_O_2_-induced increases in Mg^2+^ and decreases in ATP. **a** Fluorescence images of keratinocytes expressing the ATP sensor ATeam (CFP, YFP, DIC, and Merge). Scale bar: 100 μm. **b** Average time-courses of ATP levels in newborn keratinocytes (red line: *n* = 30 cells from four different experiments) and adult keratinocytes (blue line: *n* = 24 cells from five different experiments) in response to H_2_O_2_ (1 mM) added at 1 min. Error bars: SEM. **c** Comparison of the average amplitude of H_2_O_2_-induced decreases in ATP in the newborn and adult keratinocytes shown in **b**. The amplitude was calculated as a difference between the average of *R*/*R*_0_ before (0–1 min) and after (9–10 min) H_2_O_2_ treatment. N.S.: not significant (Student’s *t*-test, two-sided). **d** Average time-courses of ATP levels in adult keratinocytes in response to the indicated inhibitors and subsequent H_2_O_2_. These treatments were as follows: control (green line: *n* = 11 cells from three different experiments), oligomycin (blue line: *n* = 15 cells from three different experiments), and FCCP (orange line: *n* = 19 cells from three different experiments). Depending on the treatment group, oligomycin (5 μM) or FCCP (5 μM) was added at 1 min and H_2_O_2_ was subsequently added at 4 min. Error bars: SEM. **e** The average amplitude of H_2_O_2_-induced decreases in ATP in the adult keratinocytes shown in **d**. The amplitude was calculated as a difference between the average of *R*/*R*_0_ before (3–4 min) and after (12–13 min) H_2_O_2_ treatment. *p < 0.05 (Dunnett’s test, two-sided). **f** Average time-courses of Mg^2+^ response in adult keratinocytes in response to the indicated inhibitors and subsequent H_2_O_2_. These treatments were as follows: control (green line: *n* = 426 cells from three different experiments), Oligomycin (blue line: *n* = 392 cells from three different experiments), and FCCP (orange line: *n* = 398 cells from three different experiments). Depending on the treatment group, oligomycin (5 μM) or FCCP (5 μM) was added at 1 min and H_2_O_2_ was subsequently added at 4 min. Error bars: SEM. **g** The average amplitude of H_2_O_2_-induced Mg^2+^ responses in the adult keratinocytes shown in **f**. The amplitude was calculated as a difference between the average of *F*/*F*_0_ before (3–4 min) and after (12–13 min) H_2_O_2_ treatment. In the box plots in this figure, center line: median, *x*: average, box limits: quartiles, whiskers: 1.5× interquartile range. *p < 0.05 (Dunnett’s test, two-sided).

Most of the intracellular Mg^2+^ binds to various biomolecules, and a major binding partner of Mg^2+^ in the cytoplasm is ATP. ATP normally binds to Mg^2+^ in the form of an Mg–ATP complex, but the Mg^2+^ is dissociated when ATP is consumed and degraded to ADP, leading to an increase in free Mg^2+^. Mg^2+^ dissociation from ATP due to ATP consumption has been reported as the cause of increases in [Mg^2+^]_cyto_ during mitosis and apoptosis^18,32^. To confirm the dissociation of Mg^2+^ from ATP, the genetically encoded ATP sensor ATeam^33^ was expressed in keratinocytes and the intracellular ATP level was visualized (Fig. 3a). It was confirmed that H_2_O_2_ does not directly affect ATeam signals independently of ATP (Supplementary Fig. 1b). Upon an application of H_2_O_2_, ATP concentration decreased in keratinocytes (Fig. 3b blue line). To examine the relationship between the decrease in ATP and increase in [Mg^2+^]_cyto_, H_2_O_2_-induced change in ATP concentration was also examined in the newborn keratinocytes. These cells showed smaller increases in H_2_O_2_-induced [Mg^2+^]_cyto_ (Fig. 1e) and relatively smaller decreases in ATP compared to adult keratinocytes (Fig. 3b red line and c), whereas there were no significant difference in cellular ATP contents between those cells (Supplementary Fig. 4). We also examined whether the cells with large ATP decreases showed larger increases in [Mg^2+^]_cyto_. Mitochondria are a major source of cellular ATP, and their inhibition affects cellular ATP production. Therefore, the effects of prior inhibition of ATP production in the mitochondria on the H_2_O_2_-induced responses in ATP and Mg^2+^ were investigated. Neither oligomycin, which is an inhibitor of F_o_F_1_ ATP synthase, nor FCCP alone elicited decreases in ATP levels at least within minutes of the application (Supplementary Fig. 5a, b). The efficacy of oligomycin and FCCP was confirmed by the results that when combined with the glycolysis inhibitor 2-deoxy-d-glucose (2DG), those induced greater decreases in cellular ATP levels than 2DG alone (Supplementary Fig. 5c, d). These results also indicate that mitochondria and glycolysis complement each other to maintain ATP concentration in keratinocytes. Interestingly, pretreatment with oligomycin or FCCP significantly enhanced H_2_O_2_-induced decreases in ATP (Fig. 3d, e). The inhibition of ATP synthesis in the mitochondria also enhanced the H_2_O_2_-induced increase in [Mg^2+^]_cyto_, although neither oligomycin alone nor FCCP alone induced increases in [Mg^2+^]_cyto_ (Fig. 3f, g), and Mg^2+^ and ATP dynamics in response to H_2_O_2_ were mirror images of each other (compare Fig. 3d and f). These results indicate that Mg^2+^ dissociation from ATP was the major Mg^2+^ source in response to H_2_O_2_ in keratinocytes.

### Mg^2+^ suppresses the H_2_O_2_-induced decrease in MMP in a concentration-dependent manner

**Fig. 4:**
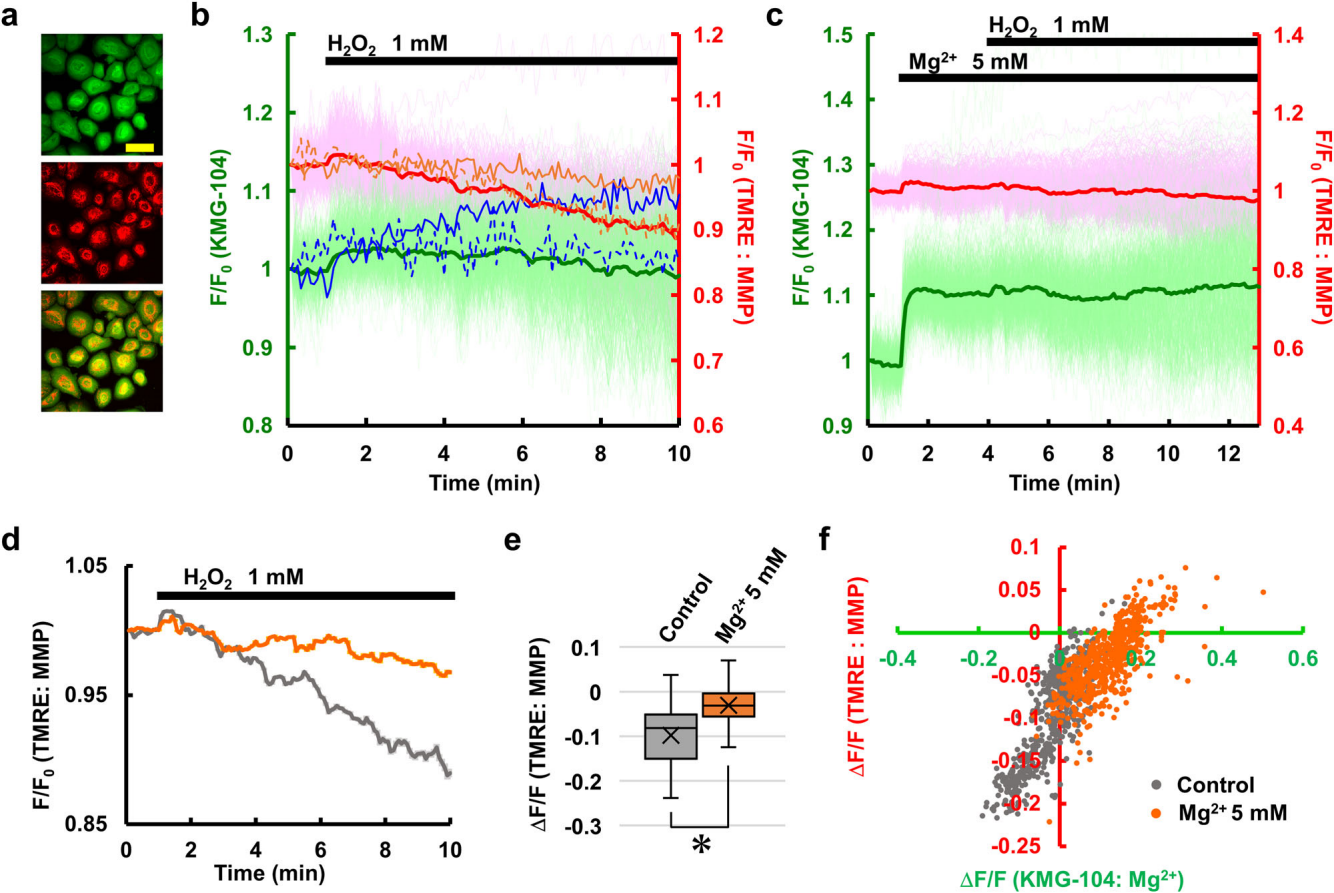
H_2_O_2_-induced decreases in MMP were suppressed by increases in Mg^2+^. **a** Fluorescence images of keratinocytes stained with KMG-104 (green) and TMRE (red), and a merged image. Scale bar: 100 μm. **b** Time-courses of [Mg^2+^]_cyto_ (green line indicates mean and light green lines indicate all traces, left axis) and MMP (red line indicates mean and pink lines indicate all traces, right axis), measured simultaneously, in response to H_2_O_2_ added at 1 min (*n* = 542 cells from three different experiments). Representative trace of normal responding cells is shown in blue ([Mg^2+^]_cyto_) and orange (MMP) dashed lines, and representative trace of cells with large Mg^2+^ responses is shown in blue ([Mg^2+^]_cyto_) and orange (MMP) solid line. **c** Time-courses of [Mg^2+^]_cyto_ (green line indicates mean and light green lines indicate all traces, left axis) and MMP (red line indicates mean and pink lines indicate all traces, right axis) in response to stepwise increases in extracellular Mg^2+^ concentration from 0.9 to 5 mM and the subsequent addition of H_2_O_2_ (*n* = 581 cells from three different experiments). **d** Average time-courses of H_2_O_2_-induced decreases in MMP under high Mg^2+^ (5 mM) conditions (orange line: *n* = 581 cells from three different experiments) and that under normal conditions (control, gray line: *n* = 542 cells from three different experiments). Error bars: SEM. **e** Comparison of the amplitudes of H_2_O_2_-induced decreases in MMP that were shown in **d**. The amplitude was calculated as a difference between the average of *R*/*R*_0_ before (0–1 min) and after (9–10 min) H_2_O_2_ treatment. Center line: median, *x*: average, box limits: quartiles, whiskers: 1.5× interquartile range. *p < 0.05. (Student’s *t*-test, two-sided). **f** Scatter plot of the H_2_O_2_-induced increases in [Mg^2+^]_cyto_ (Δ*F*/*F*_0_) and decreases in MMP (Δ*F*/*F*_0_) in each cell that were shown in **d**. These responses were measured under high Mg^2+^ conditions (orange plots) and normal conditions (gray plots).

Our next question was whether increased [Mg^2+^]_cyto_ protected cells from oxidative stress and via what mechanism this occurred. It has been reported that H_2_O_2_ suppresses mitochondrial function by decreasing MMP^5,6^. On the other hand, previous studies show that Mg^2+^ contributes to the maintenance of MMP and that increased Mg^2+^ by Mg^2+^ supplementation or inhibition of Mg^2+^ efflux attenuates decreases in MMP under cellular stress conditions^20–22^. Therefore, the relationship between [Mg^2+^]_cyto_ and MMP was examined using simultaneous imaging with KMG-104 and TMRE (Fig. 4a). H_2_O_2_ caused an increase in [Mg^2+^]_cyto_ and a gradual decrease in MMP within 10 min (Fig. 4b). Keratinocytes that showed large Mg^2+^ increases exhibited lower decreases in MMP (thin solid lines in Fig. 4b), and conversely, cells with small increases in [Mg^2+^]_cyto_ showed large decreases in MMP (thin dotted lines in Fig. 4b). To prove that Mg^2+^ had a direct effect on H_2_O_2_-induced decreases in MMP, the cytosolic level of Mg^2+^ was increased by adding Mg^2+^ to the extracellular medium before H_2_O_2_ stimulation. Supplementation of Mg^2+^ to the extracellular medium increased the Mg^2+^ concentration in the medium from 0.9 to 5 mM and led to a steep increase in [Mg^2+^]_cyto_. The subsequent application of H_2_O_2_ did not induce significant changes in averaged [Mg^2+^]_cyto_, while some cells showed an increase or decrease in [Mg^2+^]_cyto_ (green line in Fig. 4c). Interestingly, H_2_O_2_-induced decreases in MMP were significantly prevented by the prior addition of Mg^2+^ (Fig. 4d, e), whereas Mg^2+^ supplementation itself had little effect on MMP (red line in Fig. 4c). These findings suggest that [Mg^2+^]_cyto_ affects MMP primarily under stress conditions. To estimate the relationship between [Mg^2+^]_cyto_ and H_2_O_2_-induced decreases in MMP, the changes in [Mg^2+^]_cyto_ from initial levels (shown as green lines in Fig. 4b, c) and the H_2_O_2_-induced changes in MMP that were normalized at 1 min before H_2_O_2_ application (from the data in Fig. 4d) in each cell were plotted (Fig. 4f). In both the normal condition (gray dots) and the high Mg^2+^ condition (orange dots), strong correlations between Mg^2+^ increases and MMP were observed; higher levels of cytosolic Mg^2+^ were correlated with lower decreases in MMP. Interestingly, these two plots appear to line up on the same line, indicating that [Mg^2+^]_cyto_ is one of the key determinants for protecting the mitochondria from H_2_O_2_ damage (Fig. 4f).

**Fig. 5:**
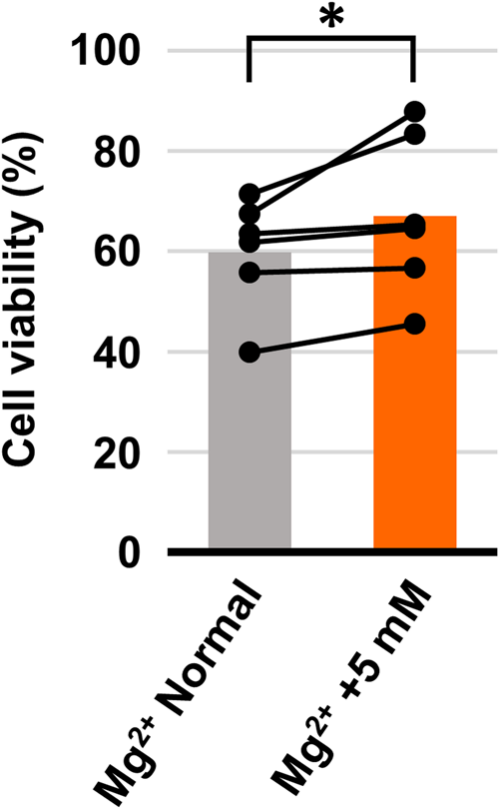
Mg^2+^ supplementation attenuated H_2_O_2_-induced cell death. Viability of the cells exposed to 1 mM H_2_O_2_ for 24 h in normal culture medium (gray) and culture medium supplemented with 5 mM Mg^2+^ (orange). Bar graphs indicate average and each pair of dots connected line indicate data of each experiment (*n* = 6). *p < 0.05. (Student’s *t*-test, one-sided).

Finally, we confirmed that the Mg^2+^ supplementation suppresses the toxicity of H_2_O_2_. Exposure to 1 mM H_2_O_2_ for 24 h induced ~40% cell death in keratinocytes in a normal culture medium. Supplementation of additional 5 mM Mg^2+^ to the culture medium suppressed the toxicity (Fig. 5). Our results indicate that Mg^2+^ supplementation is effective in protecting keratinocytes from H_2_O_2_ toxicity.

## Discussion

The skin is continuously exposed to oxidative stress. This study revealed that H_2_O_2_, which is a kind of ROS, causes increases in [Mg^2+^]_cyto_ in human keratinocytes. This H_2_O_2_-induced Mg^2+^ response was higher in adult keratinocytes than in newborn keratinocytes. Our findings indicate that the source of Mg^2+^ was dissociation from ATP in the process of ATP consumption. Upon the addition of H_2_O_2_, decreases in MMP were also observed, and the change in MMP was strongly correlated with increases in [Mg^2+^]_cyto_. In other words, higher levels of cytosolic Mg^2+^ were connected to lower MMP decreases. Furthermore, H_2_O_2_-induced decreases in MMP were significantly prevented by prior addition of Mg^2+^, suggesting direct effects of Mg^2+^ on the mitochondria. Moreover, Mg^2+^ supplementation also suppressed H_2_O_2_-induced cell death. In summary, this study revealed that H_2_O_2_-induced Mg^2+^ dissociation from ATP and that the resulting increase in [Mg^2+^]_cyto_ prevented MMP depolarization in keratinocytes. Our results suggest that Mg^2+^ that has dissociated from ATP is not merely a byproduct, but functions as a cytoprotective mechanism against oxidative stress and that Mg^2+^ supplementation is effective in protection against oxidative stress.

Mg^2+^ mobilization from intracellular storage in keratinocytes has not yet been thoroughly investigated, while Mg^2+^ influx via NIPAL4, an Mg^2+^ transporter, was reported previously^34–36^. In the present study, we demonstrated that H_2_O_2_ increases cytosolic free Mg^2+^ by dissociation from ATP due to a decrease in cellular ATP level, although neither Mg^2+^ influx, Mg^2+^ transport via Na^+^/Mg^2+^ exchanger nor Mg^2+^ release from the mitochondria was involved in this response. It has been reported that mitochondria are intracellular Mg^2+^ storage sites and that depolarization of MMP induces Mg^2+^ release from the mitochondria into the cytosol in other cell types^16,19,29,30^. In contrast, [Mg^2+^]_cyto_ instead decreased in response to FCCP in keratinocytes. This finding suggests that mitochondria do not act as Mg^2+^ sources in keratinocytes, although they probably contain Mg^2+^ in their matrix, similarly to other cell types, since some enzymes in the mitochondria require Mg^2+^ for their activity^37,38^. ATP is known to be a major intracellular Mg^2+^ binding partner: hundreds of enzymes utilize ATP in the form of Mg-ATP, and Mg^2+^ is dissociated upon the degradation of ATP into ADP, increasing intracellular free Mg^2+^^13,32,39^. Therefore, inhibition of mitochondrial ATP synthesis enhanced not only the decrease in ATP level but also the increase in [Mg^2+^]_cyto_ that was induced by H_2_O_2_. In contrast, newborn keratinocytes, which showed relatively smaller decreases in ATP compared to adult keratinocytes, had smaller Mg^2+^ responses to H_2_O_2_ than adult keratinocytes. In the process of aging, the contribution of anaerobic respiration to the energy metabolism becomes substantial in keratinocytes^40^. This difference in the ATP production process between adult and newborn keratinocytes may cause larger ATP decreases and result in greater increases in Mg^2+^ in response to H_2_O_2_ in adult keratinocytes compared to newborn keratinocytes. Our results show that keratinocytes that exhibited large decreases in ATP showed large Mg^2+^ reactions in response to H_2_O_2_ and vice versa, strongly indicating that a major source of Mg^2+^ under oxidative stress conditions is the dissociation of Mg^2+^ from ATP.

In keratinocytes, oxidative stress changes metabolism drastically and acutely. Glucose usage changes from glycolysis to the pentose phosphate pathway within seconds of oxidative stress and decreased ATP levels occurrence^11^. Our study also focused on the acute response of keratinocytes to oxidative stress and demonstrated that reduced ATP levels lead to an increase in Mg^2+^ levels. Mg^2+^ has large effects on the cellular metabolism and also on mitochondrial functions. The positive effect of Mg^2+^ on MMP under mitochondrial stress conditions has been demonstrated not only in isolated mitochondria but also in cells^20,21^. The contributions of Mg^2+^ on MMP retention via inhibiting K^+^/H^+^ exchangers, preventing mitochondrial permeability transition pores (mPTP) from opening, and activating the tricarboxylic acid (TCA) cycle, has been reported previously^41^. In the present study, MMP levels under oxidative stress were also determined by [Mg^2+^]_cyto_, which was perturbed by oxidative stress. This result suggests that the change in [Mg^2+^]_cyto_ is a mitochondria-protective mechanism under oxidative stress conditions. Previous studies have also demonstrated the long-term effects of Mg^2+^ on cell protection in keratinocytes^42^ and other cells^22,24^. [Mg^2+^]_cyto_ changes the activity of phosphatases, such as mTOR, CREB, and ERK^16^, and leads to cell protection under stress conditions in keratinocytes^42^ and neurons^22^. Therefore, Mg^2+^ has both acute and long-term protective effects for cells.

Dissociation from ATP is a well-known source of Mg^2+^. However, little has previously been known about the role of the dissociated Mg^2+^. It has been reported that an increase in [Mg^2+^]_cyto_, which is probably dissociated from Mg-ATP, is required for DNA condensation during mitosis in HeLa cells^18^, indicating that increased [Mg^2+^]_cyto_ resulting from ATP consumption is not a byproduct but instead plays an important role in cellular events. In the present study, we demonstrated that increasing levels of Mg^2+^ during the process of ATP level decrease protected the mitochondria, an organelle that is responsible for ATP generation, under oxidative stress in keratinocytes. This suggest that Mg^2+^ acts as a negative feedback signal to maintain ATP level against stress on cells. Some studies have already demonstrated that mitochondria was protected from oxidative stress under Mg^2+^ rich conditions, but these studies have not referred to changes in [Mg^2+^]_cyto_ levels^24,27,42^. Our data reveal that [Mg^2+^]_cyto_ increases in response to oxidative stress and that Mg^2+^ that dissociated from Mg-ATP is not a byproduct but instead acts as a mechanism to protect the mitochondria. Since the protective effect of Mg^2+^ supplementation against oxidative stress has been reported in other cell types^23–27^, the mechanism revealed here might be common in mammalian cells to protect cells against cellular stress.

In conclusion, we demonstrated that H_2_O_2_ induced an increase in [Mg^2+^]_cyto_ due to dissociation from Mg-ATP, and the increased [Mg^2+^]_cyto_ protected mitochondria from ROS damage. Moreover, supplementation of Mg^2+^ to extracellular medium further suppressed the decrease in MMP and attenuated H_2_O_2_ toxicity. The skin is always exposed to oxidative stress from UV, so Mg^2+^ would play an important role in maintaining the robustness of energy metabolism and protecting the skin from oxidative stress. Moreover, the addition of Mg^2+^ from an external source showed an additive effect for cell protection, suggesting that Mg^2+^ is a candidate active ingredient to protect skin from oxidative stress.

## Methods

### Cell cultures

Normal human epidermal keratinocytes were purchased from Kurabo (Osaka, Japan). To compare the response of keratinocytes from newborn babies and adults, four different batches of newborn keratinocytes vials from different donors (0 years–1, 2, 3 and 4) and two different batches of adult keratinocytes vials (donors aged 40 years and 57 years) were used (details are summarized in Supplementary Table 1). Cells were cultured in EPILIFE^TM^ medium (Thermo Fisher Scientific, Waltham, MA, USA) supplemented with insulin (10 μg mL^−1^), human recombinant epidermal growth factor (0.1 ng mL^−1^), hydrocortisone (0.67 μg mL^−1^), gentamicin (50 μg mL^−1^), amphotericin B (50 ng mL^−1^), and bovine pituitary extract (0.4%, v/v), all of which were sourced from Kurabo, at 37 °C in a CO_2_ incubator. Undifferentiated keratinocytes between passage 2 and passage 6 were used for experiments. EPILIFE^TM^ medium contains Mg^2+^, but the concentration is not disclosed.

For fluorescence imaging, keratinocytes were seeded on glass bottom dishes (IWAKI, Shizuoka, Japan) coated with 5 μg mL^−1^ collagen (Sigma-Aldrich, Saint Louis, MO, USA) at a concentration of 6–8 × 10^4^ cells mL^−1^.

### Dye loading and fluorescence imaging

For Mg^2+^ imaging, cells were stained with an Mg^2+^-selective fluorescent probe, KMG-104^43^. Keratinocytes were incubated with 20 μM KMG-104-AM and 200 μg mL^−1^ Pluronic F-127 (Thermo Fisher Scientific) at 37 °C. After 30 min, the keratinocytes were washed twice with Ca^2+^-free HBSS (Thermo Fisher Scientific; the pH was buffered using 10 mM HEPES and adjusted to 7.4 with NaOH, HBSS contains 0.9 mM Mg^2+^) and incubated for a further 15 min in Ca^2+^-free HBSS to allow the complete hydrolysis of acetoxy methyl (AM) groups. To avoid differentiation of keratinocytes, all experimental procedures were performed in Ca^2+^-free medium.

For simultaneous imaging of cytosolic Mg^2+^ and MMP, keratinocytes that had been loaded with KMG-104 were then incubated in Ca^2+^-free HBSS containing 25 nM TMRE (Thermo Fisher Scientific) for 15 min at 37 °C. Fluorescence imaging was performed in Ca^2+^-free HBSS with 2.5 nM TMRE.

A confocal laser scanning microscope system, FluoView FV1000 (Olympus, Tokyo, Japan), was used for the measurement of fluorescence. For the imaging of Mg^2+^ alone, KMG-104 was excited at 488 nm using an Ar laser through a dichroic mirror (DM405/488, Olympus), and fluorescence at 510–610 nm was detected with a photomultiplier. Images were acquired every 4–6 s. For simultaneous imaging of cytosolic Mg^2+^ and MMP, KMG-104 and TMRE were simultaneously excited at 488 nm using an Ar laser and 559 nm from a laser diode, respectively, through a dichroic mirror (DM405/488/559, Olympus). The emitted fluorescence was separated at 560 nm (SDM560, Olympus) and observed at 505–545 nm for KMG-104 and 570–670 nm for TMRE.

### Fluorescence imaging of intracellular ATP

A fluorescence resonance energy transfer (FRET)-type ATP sensor, ATeam1.03^33^, was kindly gifted from Dr. Imamura and was used to measure intracellular ATP levels. The plasmids that encoded ATeam were transfected into keratinocytes using Lipofectamine LTX (Thermo Fisher Scientific). These keratinocytes were cultured for 1–2 days after transfection to express the sensor proteins. Before observation, the cells were rinsed with and placed in Ca^2+^-free HBSS.

The cells were observed on the confocal laser scanning microscope system Fluoview FV1000. ATeam was excited at 440 nm using a laser diode through a dichroic mirror (DM405–440/515, Olympus), and the emitted fluorescence was separated by a dichroic mirror (SDM515, Olympus) and observed at 460–500 nm for CFP and at 515–615 nm for YFP.

For the negative control experiments, an ATP-insensitive variant of ATeam was constructed by inducing mutations of R122K and R126K in the ATP-sensing domain, following previous work^33^. The ATP-insensitive ATeam was expressed in the keratinocytes, and it was confirmed that H_2_O_2_ at a concentration below 10 mM had no impact on the fluorescent proteins.

### Image analysis

The acquired images were analyzed using the software packages FluoView (Olympus), Aquacosmos (Hamamatsu Photonics, Shizuoka, Japan), and ImageJ. A region of interest (ROI) was assigned to the whole cell body of each cell, and the average fluorescence intensity in each ROI was calculated respectively. After subtracting the background, the time-course of fluorescence intensity for each cell (*F*) was normalized by the initial value (*F*_0_), and the resulting *F*/*F*_0_ values were compared between KMG-104 and TMRE. For ATeam, the ratio (*R*) of the fluorescence of cyan and yellow fluorescent protein (YFP/CFP) was calculated after subtracting background. The time-course of *R* was normalized by the initial value (*R*_0_), and the resulting *R*/*R*_0_ values were compared.

Two groups of data were compared using Student’s *t*-test. To compare multiple data sets, Dunnett’s test or Tukey’s tests were used. *P* < 0.05 was used to indicate significant differences.

### Measurement of H_2_O_2_ sensitivity of KMG-104 in vitro

KMG-104 and several concentrations of H_2_O_2_ were mixed in 96-well plate and the fluorescence of these mixtures was measured using a Varioskan Flash spectral scanning multimode reader (Thermo Fisher Scientific). KMG-104 was excited at 500 nm and fluorescence intensity at 530 nm was measured and compared. H_2_O_2_ in the concentration range of 0–100 mM had no effect on the fluorescence of KMG-104 (Supplementary Fig. 1a).

### MTT assay

Keratinocytes were plated at a density of 8.0 × 10^3^ cells par well in a 96-well plate and incubated at 37 °C for more than 24 h. Medium was replaced to Mg^2+^ normal medium (normal EPILIFE with supplements) or Mg^2+^ +5 mM medium (EPILIFE with additional 5 mM Mg^2+^ and supplements) 10 min prior to H_2_O_2_ application. The medium was replaced to Mg^2+^ normal medium or Mg^2+^ +5 mM medium containing 0 or 1 mM H_2_O_2_, and cells were incubated for 24 h in the incubator. Then, the medium was replaced to 0.5 mg mL^−1^ MTT containing culture medium, and the cells were incubated for 2 h. The medium was discarded, and DMSO was then added to each well to dissolve the precipitate. The absorption at 575 nm was measured on a microplate reader, Valioscan (Thermo Fisher Scientific). The viabilities were calculated as a ratio to the average of H_2_O_2_ 0 mM condition for each Mg^2+^ concentration.

### Statistics and reproducibility

Fluorescence imaging experiments were repeated for 3–4 times for each experiment, and response of all the cells emitting sensor fluorescence in the field of view were analyzed. MTT assay was repeated for six times. Student’s *t*-test was used for comparison of a pair of data. Dannett’s test was used to compare multiple groups to control group. Tukey’s test was used to compare all differences among data group more than three groups.

### Reporting summary

Further information on research design is available in the Nature Portfolio Reporting Summary linked to this article.

### Supplementary information

Peer Review File

Supplementary Information

Description of Additional Supplementary Data

Supplementary data 1

Reporting Summary

## Data Availability

All data used in this study have been uploaded as a supplementary file named “Supplementary data 1”.

## Code availability

FluoView (Olympus), Aquacosmos (Hamamatsu Photonics, Shizuoka, Japan), and ImageJ were used for process and analyze image data.
