# Supplementary material for: Intracellular Mg2+ protects mitochondria from oxidative stress in human keratinocytes
Source: Commun Biol. 2023 Aug 24;6:868. doi: 10.1038/s42003-023-05247-6 (PMC10449934; doi:10.1038/s42003-023-05247-6)
Supplement: Supplementary file 2 — Supplementary Information [file 42003_2023_5247_MOESM2_ESM.pdf]

Supplementary Information

Intracellular Mg^{2+} protects mitochondria from oxidative stress in human keratinocytes

Keigo Fujita[1,†], Yutaka Shindo[1,2,†], Yuji Katsuta[3], Makiko Goto[3], Kohji Hotta[1], Kotaro Oka[1,2,4,5*]

[1] Department of Bioscience and Informatics, Faculty of Science and Technology, Keio University, Yokohama, Japan

[2] School of Frontier Engineering, Kitasato University, Sagamihara, Japan.

[3] MIRAI Technology Institute, Shiseido Co. Ltd., Yokohama, Japan

[4] Waseda Research Institute for Science and Engineering, Waseda University, Tokyo, Japan.

[5] Graduate Institute of Medicine, College of Medicine, Kaohsiung Medical University, Kaohsiung City, Taiwan.

[†] These authors contributed equally

Supplementary Figures

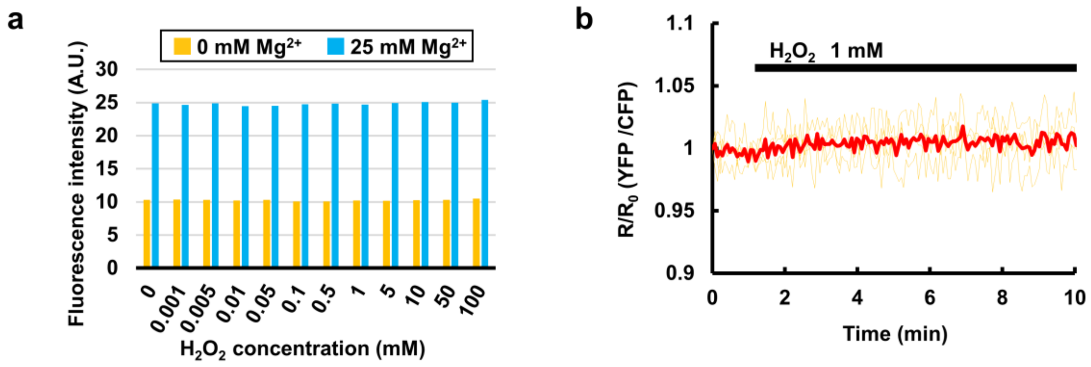

Supplementary Figure 1. Effects of H_2O_2 on fluorescent sensor molecules. (a) H_2O_2 sensitivity of KMG-104 *in vitro*. KMG-104 was mixed with the indicated concentrations of H_2O_2 in HBSS with 25 mM Mg^{2+} (blue) and Mg^{2+}-free HBSS (orange). H_2O_2 had no effect on the fluorescence of KMG-104. (b) Time-courses of the YFP/CFP ratio of keratinocytes that expressed ATP-insensitive ATeam (produced by inducing R122K and R126K mutations in the ATP-sensing domain). Red line: mean, orange lines: all traces (n = 4 cells from 3 different experiments). 1 mM H_2O_2 was added at 1 min.

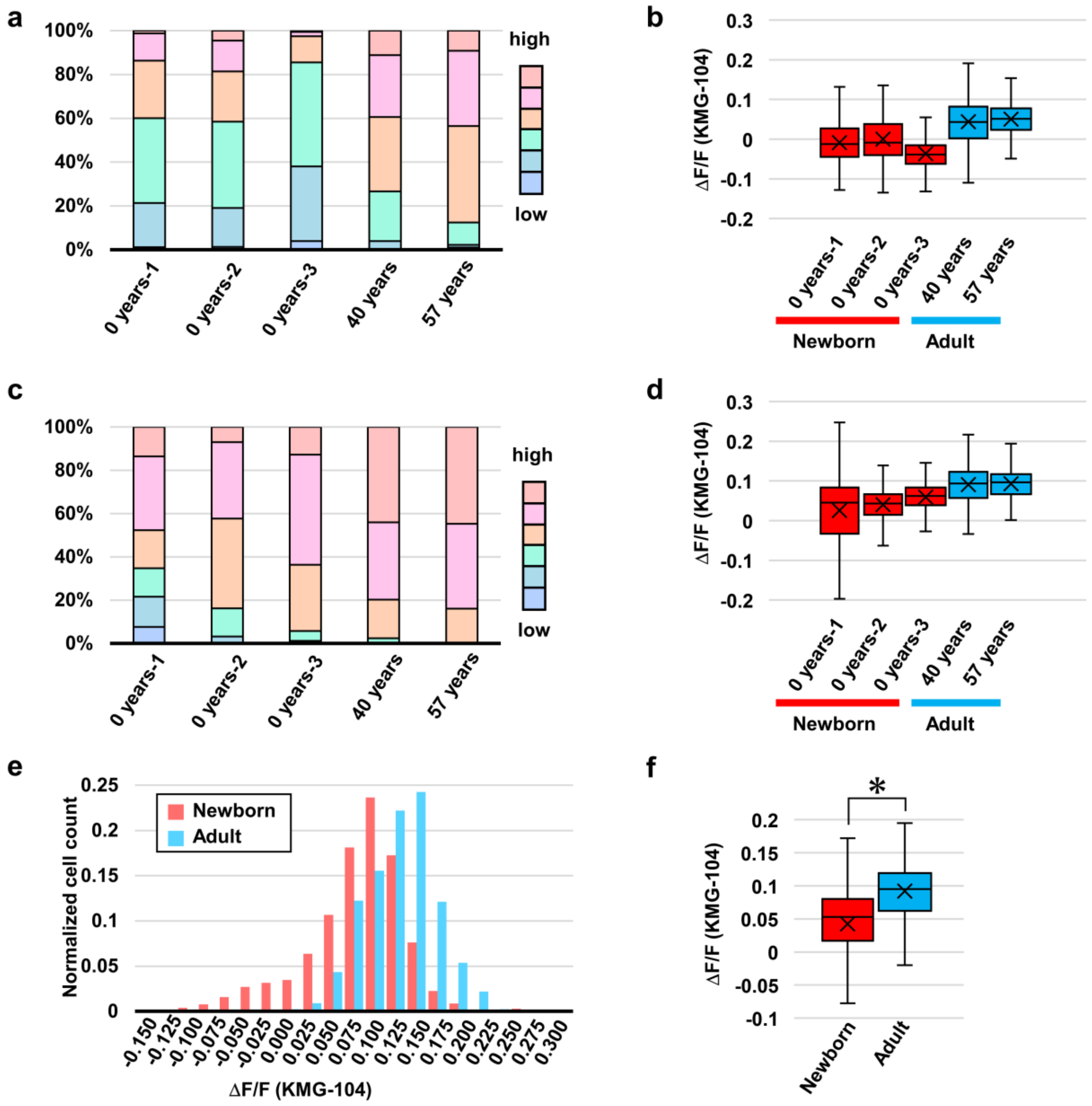

Supplementary Figure 2. Detailed analysis of H_2O_2-induced increases in $[Mg^{2+}]_{cyto}$ between age. (a) Distribution of Mg^{2+} responses induced by 1 mM H_2O_2 for each of the following batches: 3 newborn keratinocytes batches (0 years-1: n = 598 cells from 3 different experiments, 0 years-2: n = 598 cells from 3 different experiments, and 0 years-3: n = 696 cells from 3 different experiments), and 2 adult keratinocyte batches (40 years: n = 511 cells from 4 different experiments, and 57 years: n = 483 cells from 3 different experiments). Cool colors and warm colors show low and high Mg^{2+} responses, respectively. (b) Comparison of the average amplitude of 1 mM H_2O_2-induced Mg^{2+} responses in each cell line. (c) Distribution of Mg^{2+} responses induced by a high concentration (10 mM) of H_2O_2 for each of the following batches: 3 newborn keratinocytes batches (0 years-1: n = 744 cells from 3 different experiments, 0 years-2: n = 544 cells from 3 different experiments, and 0 years-3: n = 813 cells from 3 different experiments), and 2 adult keratinocyte batches (40 years: n = 370 cells from 4 different experiments, and 57 years: n = 504 cells from

3 different experiments). Cool colors and warm colors show low and high Mg^{2+} responses, respectively. (d) Comparison of the average amplitude of 10 mM H_2O_2-induced Mg^{2+} responses in each cell line. (e) Histogram showing the distribution of Mg^{2+} responses induced by a high concentration (10 mM) of H_2O_2 in newborn keratinocytes (red histogram: n = 2101 cells from 9 different experiments; the compilation of data from 0 years-1, 0 years-2 and 0 years-3) and adult keratinocytes (blue histogram: n = 874 cells from 9 different experiments; the compilation of data from 40 years and 57 years). (f) Comparison of the average amplitude of Mg^{2+} responses in response to a high concentration (10 mM) of H_2O_2 that were shown in (e). *: $p < 0.05$ (Student's t-test, two-sided). In the box plots in this figure, center line: median, x: average, box limits: quartiles, whiskers: 1.5x interquartile range.

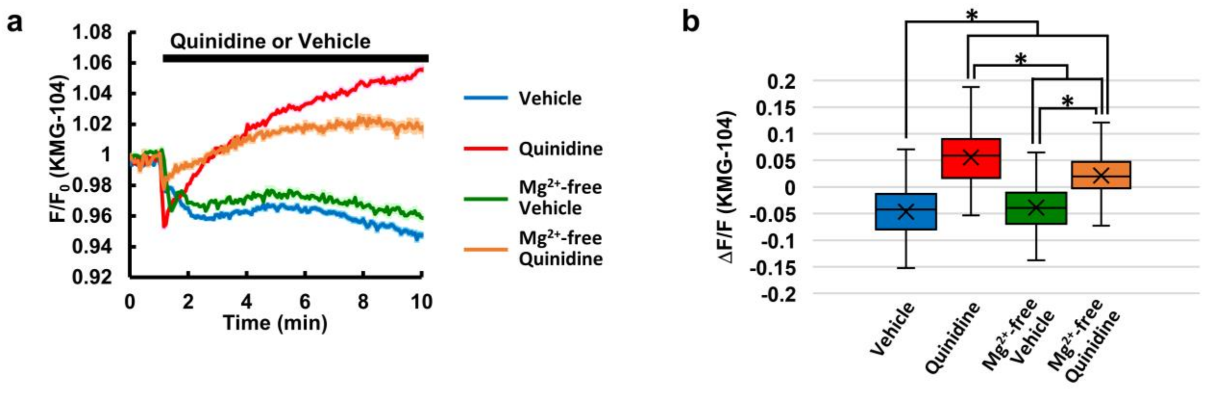

Supplementary Figure 3. Quinidine induced increases in $[Mg^{2+}]_{cyto}$

(a) Average time course of $[Mg^{2+}]_{cyto}$ in response to vehicle (DMSO, final concentration 0.5%; blue line, n = 512 cells from 4 different experiments), quinidine (200 μM; red line, n = 488 cells from 4 different experiments), vehicle in Mg^{2+}-free condition (green line, n = 340 cells from 3 different experiments), and quinidine in Mg^{2+}-free condition (orange, n = 396 cells from 3 different experiments). Quinidine or vehicle was added at 1 min by bath application. (b) Comparison of the average amplitude of Mg^{2+} response shown in (a). The amplitude was calculated as a difference between the average of F/F_0 before (0–1 min) and after (9–10 min) stimulus. Center line: median, x: average, box limits: quartiles, whiskers: 1.5x interquartile range. *: $p < 0.05$. (Tukey's test, two-sided).

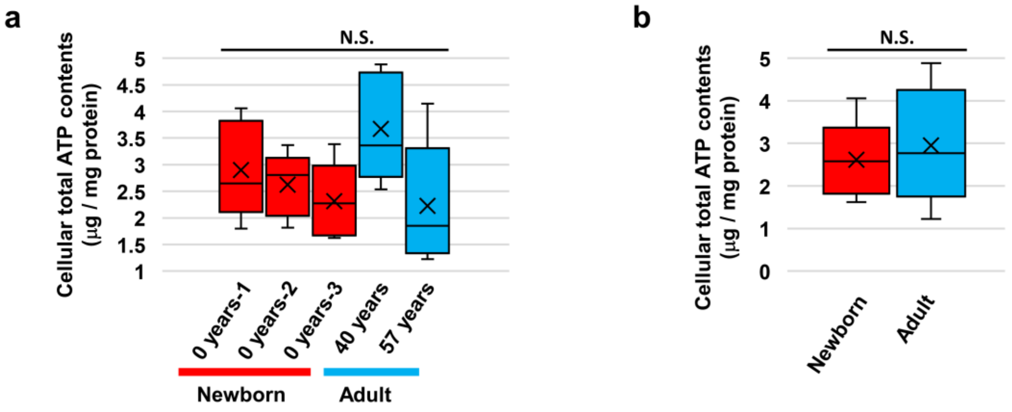

Supplementary Figure 4. ATP content in each keratinocyte line.

(a) Cellular APT contents in three newborn keratinocyte cell lines and two adult keratinocyte cell line measured by luciferin-luciferase assay (n = 5 for each). (b) Comparison of cellular ATP contents between newborn group and adult group. The data in (a) were divided into these two groups and compared. There was no significant difference (left: Tukey's test, Two-sided; right: Student's t-test, Two-sided). In the box plots in this figure, center line: median, x: average, box limits: quartiles, whiskers: 1.5x interquartile range.

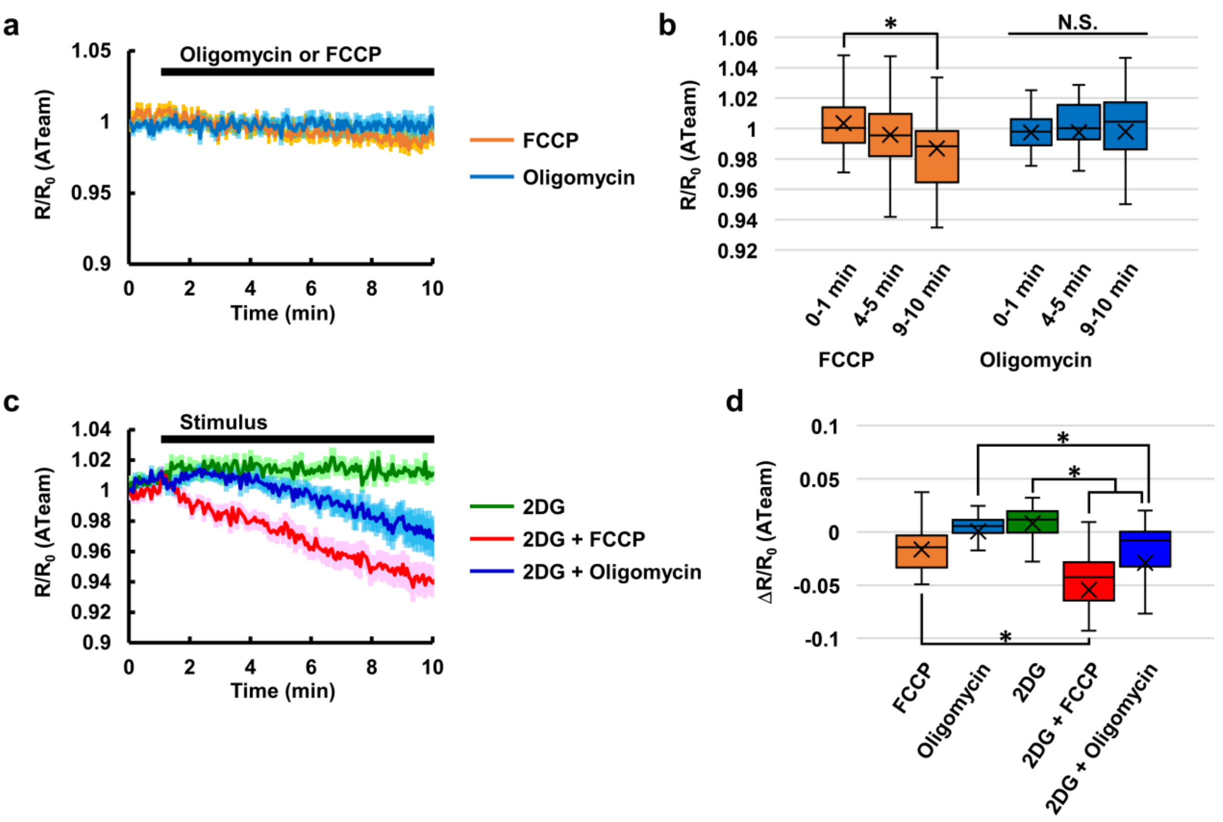

Supplementary Figure 5. Effect of oligomycin and FCCP on cellular ATP level

(a) Average time-courses ATP levels in adult keratinocytes measured with ATeam in response to FCCP (5 µM, orange line: n = 43 cells from 3 different experiments) or oligomycin (5 µM, light blue line: n = 41 cells from 3 different experiments). (b) Comparison of average ratio of the data in (a) at 0–1 min, 4–5 min, and 9–10 min. *: $p < 0.05$ (Dunnett's test, Two-sided). (c) Average time-courses ATP levels in adult keratinocytes measured with ATeam in response to 2DG (10 mM, green line: n = 32 cells from 3 different experiments), combination of 2DG and FCCP (red line: n = 34 cells from 3 different experiments) or combination of 2DG and oligomycin (blue line: n = 33 cells from 3 different experiments). The stimulus was added at 1 min. (d) Comparison of the amplitudes of responses shown in (c). The amplitude was calculated as a difference between the average of R/R_0 before (0–1 min) and after (9–10 min) stimulus. *: $p < 0.05$ (Tukey's test, Two-sided). In the box plots in this figure, center line: median, x: average, box limits: quartiles, whiskers: 1.5x interquartile range.

	Origin	Sex	Tissue source	Age	Lot number
0 years-1	Caucasian	Male	Foreskin	Neonatal	06446
0 years-2	Caucasian	Male	Foreskin	Neonatal	09185
0 years-3	Caucasian	Male	Foreskin	Neonatal	08415
0 years-4	Caucasian	Male	Foreskin	Neonatal	08344
40 years	Caucasian	Female	Abdominal	40	07510
57 years	Caucasian	Female	Abdominal	57	08138

Supplementary Table 1 Characteristics of keratinocyte cell lines. All keratinocyte cell lines purchased from Kurabo (Osaka, Japan).
