# Supplementary material for: Intracellular Mg2+ protects mitochondria from oxidative stress in human keratinocytes
Source: Commun Biol. 2023 Aug 24;6:868. doi: 10.1038/s42003-023-05247-6 (PMC10449934; doi:10.1038/s42003-023-05247-6)
Supplement: Supplementary file 3 — Description of Additional Supplementary Data [file 42003_2023_5247_MOESM3_ESM.docx]

**Description of Additional Supplementary Files**

**File name:** Supplementary Data 1

**Description:** The source data behind the graphs in the manuscript and Supplementary Figures.
